# Association of autism with polymorphisms in the paired-like homeodomain transcription factor 1 (*PITX1*) on chromosome 5q31: a candidate gene analysis

**DOI:** 10.1186/1471-2350-8-74

**Published:** 2007-12-06

**Authors:** Anne Philippi, Frédéric Tores, Jérome Carayol, Francis Rousseau, Mélanie Letexier, Elke Roschmann, Pierre Lindenbaum, Abdel Benajjou, Karine Fontaine, Céline Vazart, Philippe Gesnouin, Peter Brooks, Jörg Hager

**Affiliations:** 1 IntegraGen SA, 5 rue Henri Desbruères, Genopole Campus 1, Genavenir 8, 91000 Evry, France

## Abstract

**Background:**

Autism is a complex, heterogeneous, behaviorally-defined disorder characterized by disruptions of the nervous system and of other systems such as the pituitary-hypothalamic axis. In a previous genome wide screen, we reported linkage of autism with a 1.2 Megabase interval on chromosome 5q31. For the current study, we hypothesized that 3 of the genes in this region could be involved in the development of autism: 1) paired-like homeodomain transcription factor 1 (*PITX1*), which is a key regulator of hormones within the pituitary-hypothalamic axis, 2) neurogenin 1, a transcription factor involved in neurogenesis, and 3) histone family member Y (*H2AFY*), which is involved in X-chromosome inactivation in females and could explain the 4:1 male:female gender distortion present in autism.

**Methods:**

A total of 276 families from the Autism Genetic Resource Exchange (AGRE) repository composed of 1086 individuals including 530 affected children were included in the study. Single nucleotide polymorphisms tagging the three candidate genes were genotyped on the initial linkage sample of 116 families. A second step of analysis was performed using tightly linked SNPs covering the *PITX1 *gene. Association was evaluated using the FBAT software version 1.7.3 for single SNP analysis and the HBAT command from the same package for haplotype analysis respectively.

**Results:**

Association between SNPs and autism was only detected for *PITX1*. Haplotype analysis within *PITX1 *showed evidence for overtransmission of the A-C haplotype of markers rs11959298 – rs6596189 (*p *= 0.0004). Individuals homozygous or heterozygous for the A-C haplotype risk allele were 2.54 and 1.59 fold more likely to be autistic than individuals who were not carrying the allele, respectively.

**Conclusion:**

Strong and consistent association was observed between a 2 SNPs within *PITX1 *and autism. Our data suggest that *PITX1*, a key regulator of hormones within the pituitary-hypothalamic axis, may be implicated in the etiology of autism.

## Background

Autism spectrum disorder is a complex, heterogeneous, behaviorally-defined disorder with a 4:1 male:female gender distortion [1]. Although environmental elements, such as peri- and post-natal stress, have been reported to contribute to the development of autism, monozygotic twin studies along with evidence of chromosomal abnormalities, mutations in single genes, and multiple gene polymorphisms in autistic individuals, clearly show that autism is a largely genetic disorder [2-5].

Single mutations in neuroligin 3 and 4, cell adhesion molecules present at the post-synaptic side of the synapse, and in *SHANK3*, a scaffolding protein found in excitatory synapses, have been described in autistic individuals [6,7]. In the majority of cases, however, an overall lack of Mendelian inheritance suggests the involvement of multiple genes [5,8]. Indeed, genome-wide screens and candidate gene approaches have identified a number of chromosomal regions and genes linked with autism [9-19]. For example, a strong association between autism and *SLC25A12*, a gene encoding the mitochondrial aspartate/glutamate carrier AGC1 expressed in neurons and in neural stem cells, has been reported in 2 separate studies [17,19]. Similarly, an analysis of chromosome 16p revealed an association between autism and the protein kinase c-beta gene (*PRKCB1*), which is expressed in granule cells of the brain and B lymphocytes [20].

Although most of the genetic analyses, to date, have focused on genes expressed in the brain, the pathophysiology of autism suggests that other systems such as the immune system and the pituitary-hypothalamic axis may be involved [21,22]. In some autistic individuals, for example, abnormal secretion of pro-opio-melanocortin (POMC), adrenocorticotropin (ACTH), cortisol, and beta-endorphin has been noted [22-25].

We recently performed a genome-wide linkage scan in a group of families with autism and phrase speech delay identified in the Autism Genetic Resource Exchange (AGRE) DNA repository [20]. Among the 7 genomic regions that showed a significant increase in identity-by-descent sharing in sibling pairs with autism, 4 corresponded to regions that have previously been linked to autism (chromosome 5, 13, 16, and 17) [20]. Based on these results, in the current study, we focused on chromosome 5q31 and identified 3 genes that we hypothesized could be involved in the development of autism: 1) paired-like homeodomain transcription factor 1 (*PITX1*), which is a key regulator of hormones within the pituitary-hypothalamic axis such as ACTH, cortisol, and beta-endorphin [22,26,27]; 2) histone family member *H2AFY*, which is involved in X-inactivation in females and therefore could be a positional candidate that could explain the 4:1 male:female gender distortion present in autism [1,28]; and 3) Neurogenin 1 (*NEUROG1*), which is a transcription factor involved in neurogenesis [29]. Using single point association analyses and haplotype analyses, we found significant evidence for an association of autism with *PITX1 *but not with *H2AFY *or *NEUROG1*.

## Methods

### Subjects

Two-hundred and seventy-six families were selected from the AGRE program composed of 1086 individuals including 530 affected children. AGRE has been approved by an Institutional Review Board (IRB) to ensure the protection of research participants [30]. Each family had at least two affected children. No un-affected children were included in the study. In each family, affected siblings met the diagnostic criteria for autism according to the Autism Diagnostic Interview Revised (ADI-R) [31]. No families with known genetic defects like fragile X, RETT syndrome, or other monogenic forms of autism were included. Written informed consent was obtained from all parents included in the study. One-hundred sixteen families initially used in the linkage study were included in this study [20]. In these families, the Autism Diagnostic Observation Schedule (ADOS) evaluation was performed in 48% of the affected individuals and the concordance rate between ADI-R and ADOS was 94%.

### Candidate gene, SNP selection, and genotyping

Genes were annotated according to the 35^th ^version of the NCBI database [32]. In a first step, the three candidate genes were genotyped using on the same sample of 116 families as for the linkage study. A second step of analysis was performed using tightly linked SNPs covering gene(s) showing suggestive association at a nominal level in the first step. SNPs were genotyped according to the manufacturer's recommendations using oligoligation assays, (SNPlex) or TaqMan (Applied Biosystems, Foster City)

### Statistical analysis

The SNPs pairwise LD was evaluated by the measures of D' using the LDheatmap package. Hardy-Weinberg equilibrium (HWE) was tested in parents using the exact Chi-square statistic test [33].

Single SNP and haplotype association tests were carried out using FBAT version 1.7.3 [34]. Haplotype-specific association were also tested using the HBAT option in the FBAT package. Both single and haplotype analysis were performed using the empirical variance option to account for the linkage in the region studied. Nominal P-values were provided for each haplotype with more than ten informative families. First step analyses were not corrected for multiple testing since it was performed under a strong candidate gene hypothesis to identify potential association with the disease at the nominal level. However, p-values were corrected for multiple testing in the second step. Multiple hypothesis testing was controlled using the false discovery rate (FDR) approach proposed by Benjamini and Hochberg [35]. Because of the non-independence of tests, this method is conservative (but less so than the Bonferroni correction) and generally tends to underestimate statistical significance.

To estimate relative risk (RR) of marker(s) identified as associated to autism in the present paper, we used a conditional likelihood based method [36]. This method estimates haplotype RRs under an additive model from unphased data but also single marker like SNP risk and provides unbiased RR in case of deviation from HWE.

## Results

### Gene specific single point association analysis

**Figure 1:**
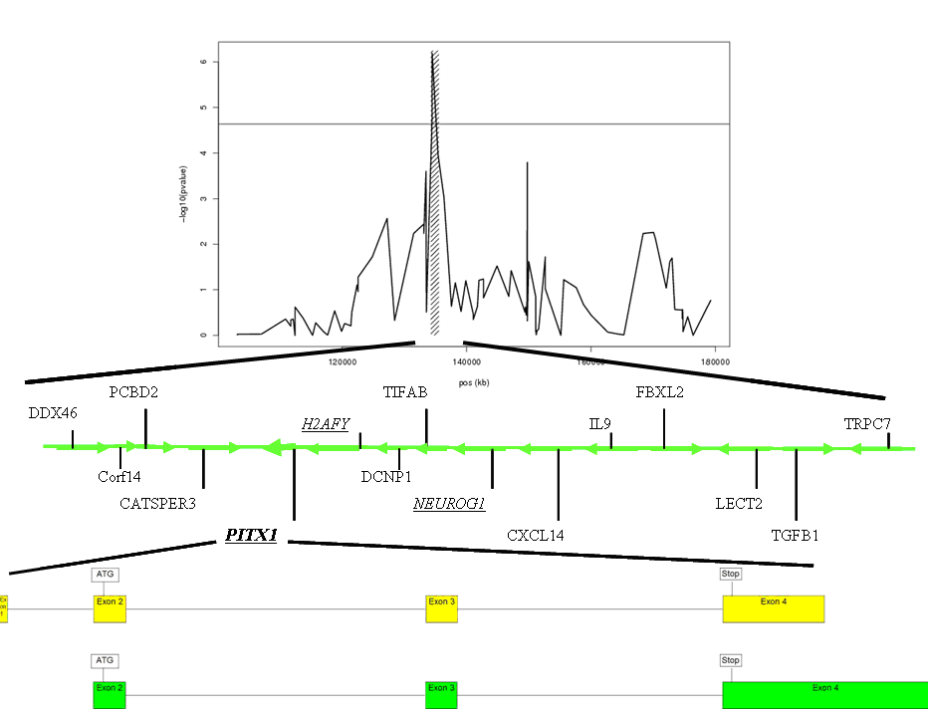
**Genomic organization of the linkage region on chromosome 5q31 and haplotype heat-map of *PITX1***. A) Five clones in the region showed elevated identity-by-descent sharing values. The highest *p*-value was observed for clone FE0DBACA4ZA08 (*p *= 6.40 * 10^-7^, position (built 34) = chr5:134.467.793 – 134.639.841). Orientation of gene transcription is indicated by arrows. The bottom panel shows the exon-intron structure of the *PITX1 *gene. Two transcripts have been described for the gene, which make use of the same start and stop codons but differ in their respective 5' and 3' UTR regions.

Of the 16 known genes in the 1.2 Mb region of chromosome 5q31 (Table 1), which was identified in a previous genome-wide linkage analysis for autism [20], 3 were selected as candidate genes based on function: *PITX1*,*H2AFY*, and *NEUROG1 *(Figure 1).

**Table 1:** Genes identified in a 1.2 Mb linkage region of chromosome 5q31

**Name**	**Product**	**NCBI GENE ID.**	**OMIM ID.**
DDX46	DEAD (Asp-Glu-Ala-Asp) box polypeptide 46	9879	NULL
Corf14	Disulfide isomerase	79770	NULL
PCBD2	Dimerization cofactor of hepatocyte nuclear	84105	NULL
CATSPER3	Cation channel, sperm associated 3	347732	609120
PITX1	Paired-like homeodomain transcription factor 1	5307	602149
H2AFY	H2A histone family, member Y isoform	9555	NULL
DCNP1	Dendritic cell nuclear protein 1	140947	NULL
TIFAB	TIFA related protein	497189	NULL
NEUROG1	Neurogenin 1	4762	601726
CXCL14	Small inducible cytokine B14 precursor	9547	604186
IL9	Interleukin 9 precursor	3578	146931
FBXL21	F-box and leucine-rich repeat protein 21	26223	609087
LECT2	Leukocyte cell-derived chemotaxin 2	3950	602882
TGFβ1	Transforming growth factor, beta-induced	7045	601692
SMAD5	SMAD, mothers against DPP homolog 5	4090	603110
TRPC7	Putative capacitative calcium channel	57113	NULL

A two step procedure was used to screen the candidate genes for association with autism. The average genotyping success rate was > 92%. All markers, except rs474853 were in Hardy-Weinberg equilibrium (Table 2). One family was removed from the analysis because of Mendelian incompatibilities.

**Table 2:** Association analyses with tag-SNPs selected in candidate genes. Single point SNP analysis results of first step analysis of *PITX1*, *H2AFY*, and *NEUROG1 *in 116 families from the initial linkage scan. Base positions are indicated according to built 35 of the human genome sequence. MAF, minor allele frequency; perm, permutations; SNP, single nucleotide polymorphism; HWE, Hardy-Weinberg equilibrium probability test. Association analysis was performed using the FBAT package.

	**SNP**	**Position (b35)**	**MAF (Allele)**	**HWE**	**Z-score (Allele)**	**P-value**
***PITX1***	rs28330	134,380,433	0.37 (T)	0.65	2.483 (C)	0.0130
	rs31210	134,388,918	0.21 (A)	1.00	1.136 (G)	0.2560
	rs474853	134,392,990	0.47 (T)	<10^-5^	*0.62 (C)*	*0.5351*
	rs3805663	134,394,099	0.31 (G)	1.00	2.741(A)	0.0061
	rs1700488	134,402,776	0.08 (A)	1.00	2.343 (G)	0.0191
***H2AFY***	rs13163460	134,692,981	0.21 (T)	0.52	1.084 (G)	0.2784
	rs6865399	134,702,478	0.10 (G)	1.00	1.294 (G)	0.1957
	rs2292011	134,707,146	0.22 (T)	0.85	1.453 (C)	0.1462
	rs1393082	134,734,972	0.39 (A)	0.89	2.004 (C)	0.0450
	rs3776203	134,736,664	0.15 (T)	0.79	1.065 (C)	0.2869
***NEUROG1***	rs6596238	134,892,355	0.39 (A)	0.55	0.769 (A)	0.4419
	rs245128	134,908,890	0.35 (A)	0.33	1.044 (G)	0.2963

Single marker analysis revealed nominally significant p-values for 3 of the 5 SNP markers selected for *PITX1*: rs28330 (*p *= 0.013), rs3805663 (*p *= 0.0061), and rs1700488 (*p *= 0.0191) (Table 2). Marker rs1700488 is positioned 4.4 kb upstream of the first exon of *PITX1*. Marker rs28330 is located 11 kb 3' from the last exon of *PITX1*. Marker rs3805663 lies between exon 3 and 4 of *PITX1*. Mutation screening by direct sequencing did not show any amino-acid or other mutational changes (data not shown).

To further investigate *PITX1 *9 SNPs, including new markers not genotyped in phase 1, were selected to fully capture the polymorphic information of the gene and genotyped in an extended family set of 276 AGRE families. Two markers showed strong significant evidence for association (rs11959298, p = 2 * 10^-4 ^and rs6596189, p = 1 * 10^-4^) and 3 other markers (rs1131611, rs6872664, rs6596188) reached significance even after correcting for multiple testing (Table 3).

**Table 3:** Extended SNP analysis in the PITX1 gene. In the second step additional markers were added in the PITX1 gene analysis. Also 160 further families were added to the analysis of *PITX1*in this step. Thus step 2 included a total of 276 families for genotyping. Markers rs28330, rs31210, rs474853, rs3805663, rs1700488, rs13163460, rs3776203, rs6596238, rs245128, rs2249596, rs1131611, rs254550, and rs254551 were genotyped using SNPlex. Markers rs6865399, rs2292011, rs1393082, rs657223, rs7700313, and rs39882 were genotyped using TaqMan, respectively. Base positions are indicated according to built 35 of the human genome sequence. MAF, minor allele frequency; perm, permutations; SNP, single nucleotide polymorphism; HWE, Hardy-Weinberg equilibrium probability test. Association analysis was performed using the FBAT package.

**SNP**	**Position (built 35)**	**MAF (Allele)**	**HWE**	**Z-score (Allele)**	**P-value (P_**corrected**_)**
rs657223	134,383,698	0.40(T)	0.21	1.287(A)	0.1980
rs479632	134,392,417	0.23 (G)	1.00	0.539 (C)	0.5896
rs1131611	134,392,894	0.12 (T)	0.41	2.433 (G)	0.0150 (0.0283)
rs888685	134,393,688	0.36 (A)	0.40	1.929 (T)	0.0537
rs3805663	134,394,099	0.36 (G)	0.22	1.425 (A)	0.1542
rs11959298	134,395,438	0.13 (G)	1.00	3.668 (A)	0.0002 (0.0017)
rs6872664	134,395,496	0.12 (T)	0.31	2.814 (C)	0.0049 (0.0167)
rs6596188	134,396,001	0.12 (T)	0.31	2.636 (A)	0.0084 (0.0178)
rs6596189	134,396,067	0.13 (T)	0.70	3.825 (C)	0.0001 (0.0017)
rs7700313	134,396,298	0.12 (T)	0.82	1.897 (T)	0.0578

### Linkage disequilibrium and haplotype analysis of *PITX1 *gene

**Figure 2:**
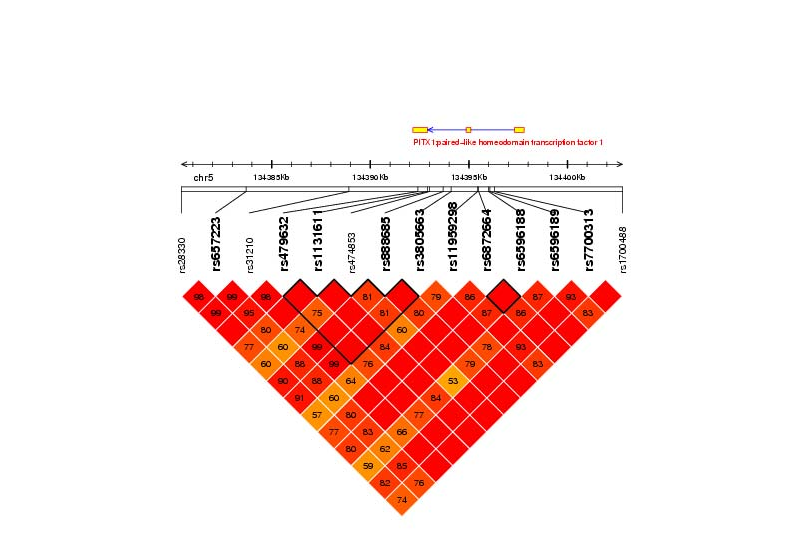
**Linkage disequilibrium (LD) map of the *PITX1*.gene**. Thirteen SNP markers within the 6.5 kb of genomic sequence span the 5' and 3' UTRs of *PITX1*. Pair-wise LD among SNPs was investigated using D'. Haplotype blocks were determined by identifying the first and last markers in a block, which are in strong LD with all intermediate markers. The structure and position of the *PITX1 *gene, the positions of the 5 SNPs from the first step genotyping and the 9 SNPS from the second step genotyping are indicated (SNP markers in bold), respectively. LD, linkage disequilibrium; SNP, small nucleotide polymorphism; UTR, 5'-untranslated regions.

Haplotypes analysis was conducted to extract more inheritance information from the set of markers. As a first step, the LD structure among the 9 markers was evaluated in *PITX1 *(Figure 2). We observed two blocks of LD, a four SNP block (block 1) and a two SNPs block (block 2). Block 1 is composed of SNPs that individually were not significant after correction for multiple testing. Grouping into haplotypes, did not increase significance after correction, apart from a slight tendency for a protective effect of haplotype C-T-A-G (Table 4). The second LD block contains two SNPs that showed significant association with p = 0.0084 (p_corrected _= 0.0178) for the two haplotype alleles having a frequency greater than 0.05). This block is bordered by two SNPs (rs11959298 and rs6596189) that present strong LD (D' = 1.0 and r^2 ^= 0.98) between them and milder LD (D' = 0.83 or 0.85) with markers from block 2 (Figure 2). They displayed the strongest significant single point association and combined into haplotypes also provided strong evidence for association with p = 0.0004 (p_corrected _= 0.0017) for haplotype A-C (Z-score = 3.530). Defining allele A-C as the risk allele and homozygous carrier of allele G-T as the reference genotype, we estimated the RR to 1.59 ([1.26 ; 2.02] 95% confidence interval) for a heterozygous carrier and 2.54 ([1.58 ; 4.09]) for homozygous carrier.

**Table 4:** Results of haplotype association analysis

SNPs ID	Alleles	Freq.	Z-score	P-value^3 ^(*P*_*corrected*_)	Relative Risk
rs479632 – rs1131611 – rs888685 – rs3805663	C-G-T-A	0.64	2.000	0.0455 (*0.0703*)	
	G-G-A-G	0.24	-0.267	0.7894	
	C-T-A-G	0.11	-2.249	0.0245 (*0.0416*)	
rs6872664 – rs6596188	C-A	0.88	2.636	0.0084 (*0.0178*)	RR_HT _= 1.46 [1.13; 1.88] RR_HO _= 2.12 [1.27; 3.54]
	T-T	0.12	-2.636	0.0084 (*0.0178*)	
rs11959298 – rs6596189	A-C	0.88	3.530	0.0004 (*0.0017*)	RR_HT _= 1.59 [1.26; 2.02] RR_HO _= 2.54 [1.58; 4.09]
	G-T	0.12	-3.630	0.0003 (*0.0017*)	

## Discussion

In this study, we have found a significant association between autism and polymorphisms of *PITX1*, a paired-like homeodomain transcription factor involved in hormonal regulation [26,27]. Using a two step procedure we initially identified evidence for association for marker rs3805663 with autism. In the second step additional markers in the PITX1 gene were genotyped in an extended sample set of 276 families total. Although in this extended set marker rs3805663 did not reach a significant p-value any more, several additional markers showed highly significant results with the most significant result for rs6596189 (p = 1 * 10^-4^). Haplotype analyses yielded a couple of highly significant pairings, although none of these showed higher significance than rs6596189 alone. Individuals homozygous or heterozygous for the risk allele were 2.69 and 1.74 fold more likely to be autistic than individuals who were not carrying the allele, respectively.

*PITX1 *is a key regulator of hormonal genes in the pituitary-hypothalamic axis. Its putative involvement in autism is supported by evidence documenting abnormal levels of hormones such as ACTH, beta-endorphin, and cortisol in autistic individuals and by the fact that these hormones are downstream of *PITX1 *[22-27]. Deregulation of POMC and high levels of beta-endorphin in the morning, for example, have been shown to be involved in certain maladaptive behaviors, such as self-injurious behaviors, which are often seen in autistic individuals [37]. The ACTH-cortisol system, which also plays an important role in stress related responses, is impaired in autistic individuals in whom lower cortisol levels and higher ACTH levels have been reported [24].

Linkage between chromosome 5q31-32 and autism is consistent with previously published studies. Both the IMGSAC (1998) genome-wide linkage scan and the screen performed by Risch and colleagues found modestly elevated LOD-scores in this region and exclusion mapping analyses did not clearly exclude this region [10,12].

Interestingly, this genomic region was recently identified as a potential locus for attention deficit/hyperactivity disorder (ADHD) [38-40]. In a genome-wide scan using large multi-generational pedigrees, Arcos-Burgos and colleagues established an exclusion map for the region and defined a critical interval from 119 to 135 Mb, which encompasses the *PITX1 *gene region [38]. Their most significant family-specific microsatellite marker D5S2117 at 133.4 Mb is less than 1 Mb from the *PITX1 *gene locus.

Mutation screening of the coding region of *PITX1 *revealed a single known mis-sense mutation with a MAF of 0.33. As the single point association results did not show any evidence for association, the mutation seems unlikely to be directly involved in susceptibility to autism. The most positive SNPs in the single marker analysis as well as the positive haplotypes are all situated in the first intron of the PITX1 gene. Regulatory site analysis shows a prediction for an alternative promoter at bases 134394831–134395400, which overlaps exactly with the region covered by the four most significant SNPs. However, to our knowledge activity of this predicted promoter has not been shown. Also these SNPs are in strong LD with SNPs within the 5' region of the PITX1 gene and the designated promoter region. However, one SNP within the PITX1 promoter, rs7700313 genotyped by us did not show significant evidence for association (p_nominal _= 0.0578, Table 3). Therefore at this stage it is not possible to postulate where the functional variant is situated and if any of the SNPs genotyped is functional.

The linkage region also covers the *H2AFY *and *NEUROG1 *loci, which in view of their function in X-chromosome inactivation and neuronal development, respectively, were considered reasonable candidates [28,29,40,41]. We found associations neither for the 5 SNPs selected for *H2AFY *nor for the 2 SNPs selected for *NEUROG1*. Moreover, mutation screening by direct sequencing did not show any amino-acid or other mutational changes within these genes.

## Conclusion

Although the mechanisms by which *PITX1 *may contribute to the susceptibility to autism are yet to be explored, the genetic association between *PITX1 *polymorphisms and autism, described here, could provide an explanation for the abnormal level of hormones of the pituitary-adrenal axis reported in the literature.

## Competing interests

All authors are salaried employees of Integragen SA.

## Authors' contributions

ER, JH, and PB were responsible for developing study concepts and design. FR, KF and CV were responsible for laboratory work such as genotyping. AP, FT, JC and ML performed the statistical analyses. AB, PG, and PL were responsible for the bioinformatics. The manuscript was approved by the authors.

## Pre-publication history

The pre-publication history for this paper can be accessed here:



## References

[B1] Chakrabarti S, Fombonne E (2001). Pervasive developmental disorders in preschool children. JAMA.

[B2] Bailey A, Le Couteur A, Gottesman I, Bolton P, Simonoff E, Yuzda E, Rutter M (1995). Autism as a strongly genetic disorder: evidence from a British twin study. Psychol Med.

[B3] Steffenburg S, Gillberg C, Hellgren L, Andersson L, Gillberg IC, Jakobsson G, Bohman M (1989). A twin study of autism in Denmark, Finland, Iceland, Norway and Sweden. J Child Psychol Psychiatry.

[B4] Gauthier J, Bonnel A, St-Onge J, Karemera L, Laurent S, Mottron L, Fombonne E, Joober R, Rouleau GA (2005). NLGN3/NLGN4 gene mutations are not responsible for autism in the Quebec population. Am J Med Genet B Neuropsychiatr Genet.

[B5] Muhle R, Trentacoste SV, Rapin I (2004). The genetics of autism. Pediatrics.

[B6] Durand CM, Betancur C, Boeckers TM, Bockmann J, Chaste P, Fauchereau F, Nygren G, Rastam M, Gillberg IC, Anckarsater H, Sponheim E, Goubran-Botros H, Delorme R, Chabane N, Mouren-Simeoni MC, de Mas P, Bieth E, Roge B, Heron D, Burglen L, Gillberg C, Leboyer M, Bourgeron T (2007). Mutations in the gene encoding the synaptic scaffolding protein SHANK3 are associated with autism spectrum disorders. Nat Genet.

[B7] Jamain S, Quach H, Betancur C, Rastam M, Colineaux C, Gillberg IC, Soderstrom H, Giros B, Leboyer M, Gillberg C, Bourgeron T (2003). Mutations of the X-linked genes encoding neuroligins NLGN3 and NLGN4 are associated with autism. Nat Genet.

[B8] Jorde LB, Hasstedt SJ, Ritvo ER, Mason-Brothers A, Freeman BJ, Pingree C, McMahon WM, Petersen B, Jenson WR, Mo A (1991). Complex segregation analysis of autism. Am J Hum Genet.

[B9] Barrett S, Beck JC, Bernier R, Bisson E, Braun TA, Casavant TL, Childress D, Folstein SE, Garcia M, Gardiner MB, Gilman S, Haines JL, Hopkins K, Landa R, Meyer NH, Mullane JA, Nishimura DY, Palmer P, Piven J, Purdy J, Santangelo SL, Searby C, Sheffield V, Singleton J, Slager S (1999). An autosomal genomic screen for autism. Collaborative linkage study of autism. Am J Med Genet.

[B10] (1998). A full genome screen for autism with evidence for linkage to a region on chromosome 7q. International Molecular Genetic Study of Autism Consortium. Hum Mol Genet.

[B11] Philippe A, Martinez M, Guilloud-Bataille M, Gillberg C, Rastam M, Sponheim E, Coleman M, Zappella M, Aschauer H, Van Maldergem L, Penet C, Feingold J, Brice A, Leboyer M, van Malldergerme L (1999). Genome-wide scan for autism susceptibility genes. Paris Autism Research International Sibpair Study. Hum Mol Genet.

[B12] Risch N, Spiker D, Lotspeich L, Nouri N, Hinds D, Hallmayer J, Kalaydjieva L, McCague P, Dimiceli S, Pitts T, Nguyen L, Yang J, Harper C, Thorpe D, Vermeer S, Young H, Hebert J, Lin A, Ferguson J, Chiotti C, Wiese-Slater S, Rogers T, Salmon B, Nicholas P, Myers RM (1999). A genomic screen of autism: evidence for a multilocus etiology. Am J Hum Genet.

[B13] Liu J, Nyholt DR, Magnussen P, Parano E, Pavone P, Geschwind D, Lord C, Iversen P, Hoh J, Ott J, Gilliam TC (2001). A genomewide screen for autism susceptibility loci. Am J Hum Genet.

[B14] Buxbaum JD, Silverman JM, Smith CJ, Kilifarski M, Reichert J, Hollander E, Lawlor BA, Fitzgerald M, Greenberg DA, Davis KL (2001). Evidence for a susceptibility gene for autism on chromosome 2 and for genetic heterogeneity. Am J Hum Genet.

[B15] (2001). A genomewide screen for autism: strong evidence for linkage to chromosomes 2q, 7q, and 16p. Am J Hum Genet.

[B16] Shao Y, Raiford KL, Wolpert CM, Cope HA, Ravan SA, Ashley-Koch AA, Abramson RK, Wright HH, DeLong RG, Gilbert JR, Cuccaro ML, Pericak-Vance MA (2002). Phenotypic homogeneity provides increased support for linkage on chromosome 2 in autistic disorder. Am J Hum Genet.

[B17] Ramoz N, Reichert JG, Smith CJ, Silverman JM, Bespalova IN, Davis KL, Buxbaum JD (2004). Linkage and association of the mitochondrial aspartate/glutamate carrier SLC25A12 gene with autism. Am J Psychiatry.

[B18] Yonan AL, Palmer AA, Smith KC, Feldman I, Lee HK, Yonan JM, Fischer SG, Pavlidis P, Gilliam TC (2003). Bioinformatic analysis of autism positional candidate genes using biological databases and computational gene network prediction. Genes Brain Behav.

[B19] Segurado R, Conroy J, Meally E, Fitzgerald M, Gill M, Gallagher L (2005). Confirmation of association between autism and the mitochondrial aspartate/glutamate carrier SLC25A12 gene on chromosome 2q31. Am J Psychiatry.

[B20] Philippi A, Roschmann E, Tores F, Lindenbaum P, Benajou A, Germain-Leclerc L, Marcaillou C, Fontaine K, Vanpeene M, Roy S, Maillard S, Decaulne V, Saraiva JP, Brooks P, Rousseau F, Hager J (2005). Haplotypes in the gene encoding protein kinase c-beta (PRKCB1) on chromosome 16 are associated with autism. Mol Psychiatry.

[B21] Sweeten TL, Posey DJ, McDougle CJ (2003). High blood monocyte counts and neopterin levels in children with autistic disorder. Am J Psychiatry.

[B22] Chamberlain RS, Herman BH (1990). A novel biochemical model linking dysfunctions in brain melatonin, proopiomelanocortin peptides, and serotonin in autism. Biol Psychiatry.

[B23] Leboyer M, Bouvard MP, Recasens C, Philippe A, Guilloud-Bataille M, Bondoux D, Tabuteau F, Dugas M, Panksepp J, Launay JM (1994). Difference between plasma N- and C-terminally directed beta-endorphin immunoreactivity in infantile autism. Am J Psychiatry.

[B24] Curin JM, Terzic J, Petkovic ZB, Zekan L, Terzic IM, Susnjara IM (2003). Lower cortisol and higher ACTH levels in individuals with autism. J Autism Dev Disord.

[B25] Tordjman S, Anderson GM, McBride PA, Hertzig ME, Snow ME, Hall LM, Thompson SM, Ferrari P, Cohen DJ (1997). Plasma beta-endorphin, adrenocorticotropin hormone, and cortisol in autism. J Child Psychol Psychiatry.

[B26] Lamonerie T, Tremblay JJ, Lanctot C, Therrien M, Gauthier Y, Drouin J (1996). Ptx1, a bicoid-related homeo box transcription factor involved in transcription of the pro-opiomelanocortin gene. Genes Dev.

[B27] Hiroi N, Kino T, Bassett M, Rainey WE, Phung M, Abu-Asab M, Fojo T, Briata P, Chrousos GP, Bornstein SR (2003). Pituitary homeobox factor 1, a novel transcription factor in the adrenal regulating steroid 11beta-hydroxylase. Horm Metab Res.

[B28] Mermoud JE, Costanzi C, Pehrson JR, Brockdorff N (1999). Histone macroH2A1.2 relocates to the inactive X chromosome after initiation and propagation of X-inactivation. J Cell Biol.

[B29] Ma Q, Fode C, Guillemot F, Anderson DJ (1999). Neurogenin1 and neurogenin2 control two distinct waves of neurogenesis in developing dorsal root ganglia. Genes Dev.

[B30] Autism Genetic Resource Exchange.

[B31] Lord C, Rutter M, Le Couteur A (1994). Autism Diagnostic Interview-Revised: a revised version of a diagnostic interview for caregivers of individuals with possible pervasive developmental disorders. J Autism Dev Disord.

[B32] National Center for Biotechnology Information (NCBI).

[B33] Wigginton JE, Cutler DJ, Abecasis GR (2005). A note on exact tests of Hardy-Weinberg equilibrium. Am J Hum Genet.

[B34] Rabinowitz D, Laird N (2000). A unified approach to adjusting association tests for population admixture with arbitrary pedigree structure and arbitrary missing marker information. Hum Hered.

[B35] Benjamini Y, Drai D, Elmer G, Kafkafi N, Golani I (2001). Controlling the false discovery rate in behavior genetics research. Behav Brain Res.

[B36] Carayol J, Philippi A, Tores F (2006). Estimating haplotype relative risks in complex disease from unphased SNPs data in families using a likelihood adjusted for ascertainment. Genet Epidemiol.

[B37] Sandman CA, Touchette P, Marion S, Lenjavi M, Chicz-Demet A (2002). Disregulation of proopiomelanocortin and contagious maladaptive behavior. Regul Pept.

[B38] Arcos-Burgos M, Castellanos FX, Pineda D, Lopera F, Palacio JD, Palacio LG, Rapoport JL, Berg K, Bailey-Wilson JE, Muenke M (2004). Attention-deficit/hyperactivity disorder in a population isolate: linkage to loci at 4q13.2, 5q33.3, 11q22, and 17p11. Am J Hum Genet.

[B39] Fisher SE, Francks C, McCracken JT, McGough JJ, Marlow AJ, MacPhie IL, Newbury DF, Crawford LR, Palmer CG, Woodward JA, Del'Homme M, Cantwell DP, Nelson SF, Monaco AP, Smalley SL (2002). A genomewide scan for loci involved in attention-deficit/hyperactivity disorder. Am J Hum Genet.

[B40] Hebebrand J, Dempfle A, Saar K, Thiele H, Herpertz-Dahlmann B, Linder M, Kiefl H, Remschmidt H, Hemminger U, Warnke A, Knolker U, Heiser P, Friedel S, Hinney A, Schafer H, Nurnberg P, Konrad K (2006). A genome-wide scan for attention-deficit/hyperactivity disorder in 155 German sib-pairs. Mol Psychiatry.

[B41] Ma DQ, Whitehead PL, Menold MM, Martin ER, Ashley-Koch AE, Mei H, Ritchie MD, Delong GR, Abramson RK, Wright HH, Cuccaro ML, Hussman JP, Gilbert JR, Pericak-Vance MA (2005). Identification of significant association and gene-gene interaction of GABA receptor subunit genes in autism. Am J Hum Genet.

